# Exploring emotion dysregulation in adolescence and its association with social immaturity, self-representation, and thought process problems

**DOI:** 10.3389/fpsyg.2024.1320520

**Published:** 2024-07-23

**Authors:** Stefania Cristofanelli, Silvia Testa, Eleonora Centonze, Giorgia Baccini, Federico Toniolo, Vincenza Vavalle, Laura Ferro

**Affiliations:** ^1^Department of Human and Social Sciences, University of Aosta Valley, Aosta, Italy; ^2^Studio Associato RiPsi, Milan, Italy; ^3^TIARÉ, Association for Mental Health, Turin, Italy

**Keywords:** emotion dysregulation, personality, adolescence, Rorschach CS, Difficulties in Emotion Regulation Scale (DERS)

## Abstract

**Background and aims:**

This study aimed to explore the complex phenomenon of emotional dysregulation, particularly in adolescence, which is associated with many mental health disorders and problems. Increasing the knowledge of clinicians and researchers in this area can be helpful in guiding future treatment plans. The aim of the study was to investigate, from an exploratory perspective, which structural aspects of adolescent functioning (assessed using the Rorschach test and administered and scored according to the Comprehensive System, CS, by Exner) were associated with different dimensions of emotional dysregulation (evaluated using the Difficulties in Emotion Dysregulation Scale, DERS).

**Method:**

Secondary data were used for the study, which included 100 adolescents, with 50 in the clinical group (patients with complex trauma histories residing in therapeutic and socio-rehabilitative communities) and 50 in the nonclinical group (recruited from a scout group and middle and high schools). The two groups were compared on terms of the mean scores obtained in the DERS scales (one-tailed t-test) and the proportions of cases that obtained pathological values for selected Rorschach CS indicators (z-test). Partial correlations were calculated between the DERS scales and the Rorschach CS variables to explore which structural dimensions of functioning were associated with different characteristics of emotional dysregulation.

**Results:**

The results indicated that the two groups differed in their outcomes on all DERS scales, except for Awareness and Goals, and on four Rorschach CS variables (EgoIndex, a:p, Wsum6, and MOR). Some significant positive and negative correlations between the Rorschach CS variables and the DERS scales also emerged.

**Conclusion:**

These results suggest that the dimensions of functioning associated with emotional dysregulation are related to self-representation, relational immaturity, and thought processes character and characterize membership in a therapeutic community. The correlations described in the article warrants further consideration. Finally, the study’s limitations and future research prospects are presented.

## 1 Introduction

Defining emotional dysregulation unequivocally is a challenging task. The literature contains multiple definitions (D’Agostino et al., 2017) and the starting point for conceptualizing it is understanding the complementary construct of emotional regulation. For instance, Gross (1998) defines emotional regulation as an attempt to change the type of emotion, the timing of its experience, and the way it is expressed. According to Gross and Jazaieri (2014), emotional dysregulation occurs when there is a failure or an improper use of strategies to modulate affect. In our study, we adopted the model proposed by Gratz and Roemer (2004), which identifies six dimensions of emotional dysregulation:

1. Non-acceptance of Negative Emotions (Nonacceptance): inability to accept negative emotions, leading to discomfort or secondary negative emotions in response to emotional reactions triggered by a situation.
2. Difficulty in Achieving Goals (Goals): difficulties in concentration and in task completion when experiencing negative emotions.
3. Impulse Control Difficulties in the Presence of Negative Emotions (Impulse): difficulties in controlling impulses when negative emotions are present.
4. Difficulty in Attending to and Recognizing One’s Emotions (Awareness): inability to pay attention to one’s emotions and to recognize them.
5. Limited Access to Emotional Regulation Strategies and the Belief That Emotion Response is Unmodifiable Once It Occurs (Strategies): limited available strategies for emotional regulation and the belief that once an emotion has manifested, its response cannot be altered.
6. Emotional Clarity and the Inability to Identify Emotions (Clarity): lack of emotional clarity and incapacity to identify emotions.

The uniqueness of this approach lies in describing emotional dysregulation as a broad and multifaceted construct. This implies the possibility that different dimensions of emotional dysregulation are associated differently with various mental disorders. For example, Garofalo et al. (2018)—referring to Gratz and Roemer’s theoretical model—observed that different dimensions of the construct were associated with different personality disorders. Beyond these specificities, it is known that emotional dysregulation increases the risk of psychopathology (Dimaggio et al., 2017; Garofalo and Wright, 2017; Garofalo and Neumann, 2018). For example, in adolescence emotion dysregulation increases the risk of developing various mental disorders (Turpyn et al., 2015), including personality disorders and in particular borderline disorder (Fossati et al., 2014a), while in adulthood it contributes to the onset of personality disorders (Dimaggio et al., 2017), alcohol abuse (Garofalo and Velotti, 2015), self-harm (Garofalo and Wright, 2017) and interpersonal violence (Velotti et al., 2014; Garofalo et al., 2018).

Furthermore, it must be considered that emotional regulation is not a predetermined and immutable skill but a competence that develops throughout the lifespan. During the developmental period, emotion regulation is learned in different contexts such as family, school, work, as well as in different ways, via modeling, imitation and co-regulation with attachment figures or peers (Turpyn et al., 2015; Silvers, 2022). Furthermore, the development of specific regulation skills is influenced by individual factors related to temperament and the level of physiological and psychological maturity attained at each developmental stage (Siegel, 2014).

Adolescence is a stage characterized by significant neurobiological changes that also have repercussions on the emotional sphere. For example, compared to other life stages, adolescence is marked by a lower availability of dopamine, which may be responsible for the typical boredom experienced by the adolescents. Simultaneously, teens’ experience a greater release of dopamine when they are engaged in rewarding actions, such as risky, impulsive, or addictive behaviors (Gazzillo, 2021). Furthermore, some of the brain regions underlying emotional regulation, such as the prefrontal cortex, are still maturing, making emotional regulation less efficient in adolescence (Ahmed et al., 2015). Collectively, these data suggest that the adolescent brains is “more emotional” than adults’ and children’s brain but less capable of modulation (Gazzillo, 2021). At the same time, it would be wrong to view adolescence as a pathological period. In fact, according to Offer et al. (1998), epidemiological data on the mental health and pathology observed in this population are comparable to those observed in adulthood. This means that most adolescents manage to fulfill the developmental tasks of this phase (e.g., separating from parents, dealing with typical psychophysical changes; Silvers, 2022). These data suggest that it is possible to distinguish between psychopathology and physiological adolescent transition (Biberdzic et al., 2018).

It may be hypothesized that when difficulties in emotional regulation are substantial and associated with other disharmonious characteristics of the adolescent’s emergent personality, this implies a degree of psychopathology that requires an inpatient treatment.

Personal functioning is multidimensional and has to do with the set of peculiar ways in which the individual interprets and reacts to life events. In addition to affect, personological functioning includes cognitive, relational and identity processes (Self). Among these, some act at an implicit level and are best captured by so-called performance-based tests, such as the Rorschach method, while others operate at a manifest level and are best captured by self-report tools (Abbate and Andraos, 2019). The Rorschach test remains one of the most used tools for evaluating personological functioning in the clinical field, due to the unique information, of which the individual is often unaware (Gritti, 2020), that the test is able to offer about the peculiar way in which the subject perceives life events, expresses and processes emotions, manages stress and shapes the image of him-or her-self, others and relationships (Abbate and Porcelli, 2017). This definition is also valid for adolescents; in fact, although the main classification manuals of mental disorders such as the DSM and the ICD recommend a certain caution in the diagnosis of personality disorders during adolescence, in most cases adolescents show an emerging personality style which is relatively organized and which characterizes the structure of their thoughts, feelings, behaviors, and tendencies (Malone and Malberg, 2017). Moreover, both pathological and physiological personality traits tend to stabilize during adolescence (Hamlat et al., 2020); indeed, the 20% or so of adolescents who display early signs of personality disorders are at higher risk of negative outcomes in adulthood (including suicide attempts, violent and criminal behavior, and interpersonal conflict), which confirms the importance of assessing personality at this life stage also (Cohen et al., 2005).

The Rorschach is an appropriate tool for the assessment of personological functioning in adolescents also. In fact, the task offers an opportunity to observe in a structured and standardized way the behavior of the adolescent who, faced with a predefined sequence of stimuli, must answer the question “What could it be?.” In this way, the adolescent displays *in vivo* a sample of his or her own problem-solving strategies. Based on the information collected during the administration and interpretation of the test, the clinician can make inferences about the ways in which the adolescent understands, represents and attributes meaning to complex environmental experiences (Meyer and Erdberg, 2018).

The present research, albeit from an exploratory perspective, had the ambitious objective of contributing to the understanding of how emotional dysregulation in adolescence is associated with personological functioning. In fact, a deeper understanding of how emotional dysregulation manifests in adolescence and how it is associated with personal functioning can enhance clinical intervention. On the one hand, it can help to identify dimensions of emotional dysregulation that may suggest the presence of concurrent mental disorders and/or a high risk of developing them. On the other hand, it can guide prevention efforts (when emotional dysregulation is not associated with clear psychopathology) and treatment interventions (when emotional dysregulation is one of the many manifestations of a psychopathology). In other words, attending to personality characteristics and emotional regulation skills can be useful in distinguishing between age-appropriate physiological challenges (Siegel, 2014) and an early warning sign of emerging psychopathology (McLaughlin et al., 2011) and this is relevant for planning both prevention and treatment interventions. In fact, recognizing the early signs of emotional dysregulation can guide prevention efforts, for example by showing caregivers what signs of distress they need to pay attention to in their teenagers to prevent the onset of a full-blown disorder. On the other hand, when emotional dysregulation reaches pathological levels, it is advisable to plan a clinical intervention that takes these characteristics into account.

Specifically, we set out to investigate which structural aspects of adolescent functioning, assessed using the Rorschach test administered and scored according to Exner’s Comprehensive System (CS; Lis et al., 2007), were associated with various dimensions of emotional dysregulation, assessed using the Italian version of the Difficulties in Emotion Dysregulation Scale (DERS; Sighinolfi et al., 2010). In particular, we aimed to:

1. Verify whether the clinical and the nonclinical groups differed in terms of the scores obtained on the DERS and the Rorschach CS variables. In particular, we expected that the participants in the clinical group would obtain higher scores on DERS than the nonclinical group and that they would reach a pathological threshold on a greater number of Rorschach CS variables than the individuals in the control group;
2. Understand which aspects of personality structure, as assessed with the Rorschach CS, were associated with difficulties in emotion regulation as assessed by the DERS.

## 2 Materials and methods

### 2.1 Sample and procedure

This study drew on secondary data provided anonymously. They were originally collected by the Tiaré Association, Mental Health Services, an association offering mental health services intended for treating adolescent psychopathology.

The sample consisted of 100 Italian adolescents (Mean Age = 14.66; Standard Deviation (SD) = 1.635) divided into two groups:

- Nonclinical: 50 participants, including 32% females and 66% males, recruited from a scout group and middle and high schools (Mean Age = 14.32; min = 12 years old; max = 17 years old; SD = 1.60. Average Level of Education = 8.76; SD = 1.79). There were no exclusion criteria precluding participation in the study.
- Clinical: 50 participants, including 36% females and 64% males (Mean Age = 15.00; min = 11 years old; max = 18 years old; SD = 1.69. Average Level of Education = 7.79; SD = 1.70), residing in therapeutic or socio-rehabilitative communities affiliated to the Tiaré Association, which is made up of a group of psychologists, psychotherapists, psychiatrists and child neuropsychiatrists specializing in the treatment of mental health disorders in adolescence and young adulthood. The adolescents who participated in the study had been in the communities for at least 3 months and they had a history of complex trauma caused by physical and psychological violence, sexual abuse and other early adverse childhood experiences, as reported in their medical history. Furthermore, they displayed a heterogeneous psychopathological profile and they shared a difficulty in regulating their emotions, as shown by their behaviors and clinical observations.

The Rorschach CS and DERS scale were administered in one sitting both for the clinical and the nonclinical group; Rorschach CS was administered first because it is the more demanding task. Moreover, the administration of a self-report might have been emotionally alarming for some adolescents, generating a mental state that could have affected the administration of the Rorschach test (Meyer et al., 2015).

In the clinical group, the administration of both the Rorschach CS and DERS Scale took place as part of the psychological assessment process to which all minors who enter the community are subjected. The psychological testing took place on site at the residential center. The aim of the testing was to provide the clinical team with useful information about the adolescents’ functioning and symptoms with a view to devising “tailored” treatment plans for them. The assessment procedure for the adolescents of the nonclinical group, instead, took place at the offices of the Tiaré Association—Services for Mental Health.

The clinical group was also assessed using SWAP-200, a clinician-report to be completed by practitioners who are already familiar with the adolescents. Notably, the protocol for using SWAP-200 requires that the clinician must have previously held at least five interviews with the adolescent (Shedler et al., 2014).

To gain a better understanding of the diagnostic heterogeneity characterizing the clinical group, the results obtained from the Shedler and Westen Assessment of Personality—200—Adolescents (SWAP-200-A; Westen et al., 2005; Shedler et al., 2014) are reported below. The SWAP-200-A is a tool consisting of 200 items completed by the clinician, allowing for the assessment of an adolescent’s personality profile.

Based on the results obtained from the SWAP-200-A, we may describe the clinical group as follows:

1. Eighteen adolescents in the clinical group could be characterized as having a non-specific personality disorder. They were not diagnosed with any specific personality disorder (PD scores ≤ 60 T), but they did not fit the high-functioning prototype either (PD scores ≤ 60 T).
2. Sixteen adolescents obtained a PD scores of over 60 T. Specifically:
  - Twelve of them received two diagnoses and a high-functioning personality score of under 60 T. The most common comorbidity being borderline personality disorder and histrionic personality disorder (4 cases), followed by histrionic and antisocial (3 cases), and antisocial and narcissistic (3 cases). All of these comorbidities involved two personality disorders from cluster B. Finally, two adolescents had comorbidities between schizoid and schizotypal personality disorder, both of which belong to cluster A.
  - Two adolescents reached the cut-off for diagnoses in each of the four personality disorders composing cluster B (narcissistic, histrionic, borderline, antisocial).
  - Two adolescents received three diagnoses from cluster B (antisocial, histrionic, narcissistic personality disorder; antisocial, borderline, histrionic personality disorder).
3. One adolescent reached the clinical cut-off for histrionic personality disorder (T score ≥ 60) but also fit the prototype of a healthy personality (T score ≥ 60). According to the clinical guidelines presented in the Manual, this profile cannot be classified as a personality disorder.
4. Thirteen adolescents received a single diagnosis (PD scores ≥ 60 T and high-functioning score ≤ 60 T): five received histrionic personality disorder diagnosis, four antisocial personality disorder, and four narcissistic personality disorder.
5. The majority of diagnoses were from cluster B personality disorders (14 antisocial, 17 histrionic, 7 borderline, and 6 narcissistic); only a few diagnoses were from cluster A (6 schizotypal and 2 schizoid).
6. Two adolescents fit the high-functioning personality style (T scores ≥ 60).

The psychological testing was conducted by a team of psychologists and/or neuropsychiatrists who work in the host community and have over 15 years of experience.

### 2.2 Instruments

#### 2.2.1 Difficulties in emotion regulation scale

Difficulties in Emotion Regulation Scale (DERS; Gratz and Roemer, 2004): the DERS is a self-report questionnaire comprising 36 items rated on a 5-point Likert scale (ranging from 1 = almost never to 5 = almost always), designed to assess patterns of emotional dysregulation. The scale includes a global score and six scales: non-acceptance (Cronbach’s α = 0.84), which evaluates difficulty in accepting one’s negative emotions; goals (Cronbach’s α = 0.73) captures difficulty in achieving goals; impulse (Cronbach’s α = 0.87), which measures difficulties in controlling impulses; awareness (Cronbach’s α = 0.62), which refers to the difficulties in paying attention and recognizing emotions; strategies (Cronbach’s α = 0.84), which assesses the lack of emotion regulation strategies; and clarity (Cronbach’s α = 0.74), which captures the degree to which the individual can correctly distinguish among their emotions.

In our study, we used the validated Italian version of the DERS, as validated by Sighinolfi et al. (2010) in an adult sample. In that study, the scale scores displayed sufficient convergent, discriminant and criterion validity when compared with other self-report instruments used to measure anxiety, depression and positive and negative affect (Sighinolfi et al., 2010). Regarding the adolescent age group, Neumann et al. (2010) explored the utility of using the DERS in a sample of 870 young people aged from 11 to 17 years old. Their results displayed internal consistency and validity (Neumann et al., 2010). Such validation studies for the DERS do not exist in Italy, however many Italian researchers employ this scale to evaluate emotional dysregulation in adolescents (Fossati et al., 2014a,b; Pace et al., 2016).

#### 2.2.2 Rorschach comprehensive system

The Rorschach Comprehensive System (CS) is a performance-based test composed by a set of 10 standardized inkblots for assessing an individual’s personality, emotional functioning, and thought processes. The Rorschach CS method, which was developed by John Exner in the 1970s, has since become one of the most widely used and researched projective tests in clinical and forensic psychology. During the administration of the Rorschach CS test, the clinician presents the inkblots one at a time and asks the participant: “What could it be?.” The participant’s responses are recorded and later analyzed using a set of scoring procedures to identify specific characteristics of the participant’s personality and emotional functioning (Parolin and di Lorenzo, 2009). We utilized the computerized ROR-SCAN program (copyright 1988–2014 by Philip F. Caracena) to obtain a structural overview and an interpretative report for each protocol. We selected specific CS variables based on their clinical significance and empirical evidence. In particular, we started from the list of variables selected by Mihura et al. (2013) in their meta-analytic work aimed at identifying Rorschach CS variables with stronger empirical support. From that list, two expert clinicians (15 years of experience) in the administration and interpretation of the Rorschach Comprehensive System selected the variables that best represented the emotion dysregulation construct based on the clinical meaning of the variables (Exner, 2003; Lis et al., 2007; Abbate and Porcelli, 2017) and based on their clinical experience. In total, we retained the 23 variables selected by both the clinicians. These are described in Appendix.

### 2.3 Data analysis

Before proceeding with the statistical analyses, the Rorschach CS variables were transformed into dummy variables (0 = non-pathological score and 1 = pathological score), according to the clinical cut-off defined in Appendix. For each of the Adj.es, EgoIndex, Blends, and FM variables, two sets of dummy variables (suffix A and B) were constructed given that very high or very low scores on these variables serve as markers of emotional dysregulation.

To test whether the clinical group exhibited higher levels of emotional dysregulation compared to the nonclinical group, the means of the DERS scores between the two groups were compared using a one-tailed t-test, with Cohen’s d used to estimate effect size. To test whether the proportion of pathological identified for each Rorschach variable differed between the two groups, the proportions test (z-test) was conducted, with Cohen’s h used to estimate effect size.

Finally, partial correlations were computed, while controlling for group membership, between the DERS scales and the Rorschach CS variables. All analyses were conducted using SPSS software (version 29).

## 3 Results

### 3.1 Comparison of mean scores of DERS scales across the two groups

Table 1 presents the comparison of the mean scores on the DERS subscales obtained by the clinical and nonclinical groups, respectively.

**Table 1:** Student’s t outcomes for the differences in mean DERS scores across the clinical and nonclinical groups.

	Clinical group		Nonclinical group		Student’s t	df	*p*-value one tail	Cohen’s d
	M	SD	M	SD				
Nonacceptance	15.2	6.5	12.1	4.6	2.770	88.1	0.003	0.554
Goals	16.3	5.1	15.6	4.4	0.740	98.0	0.231	0.148
Impulse	17.3	6.9	13.8	4.7	2.941	86.2	0.002	0.588
Awareness	15.5	5.6	15.2	4.0	0.390	89.1	0.349	0.078
Strategies	21.3	8.6	17.2	6.6	2.686	91.3	0.004	0.537
Clarity	12.5	4.8	10.8	3.6	1.985	91.7	0.025	0.397
Total scale	98.1	28.1	84.6	18.9	2.812	86.0	0.003	0.562

df, degrees of freedom; M, Mean; SD, Standard Deviation.

The clinical group obtained higher scores compared to the nonclinical group on the Nonacceptance, Impulse, Strategies, Clarity, and total score of the DERS scales. In terms of effect size, the difference between the two groups was of moderate magnitude (Cohen’s d around 0.5). No statistically significant differences were observed in relation to the Goals and Awareness scales.

### 3.2 Comparison of Rorschach CS variables across the two groups

Table 2 presents the differences between the proportion of adolescents obtaining a pathological score in the selected Rorschach CS variables of the clinical and nonclinical groups.

**Table 2:** Rorschach CS variables: proportions of pathological scores for the clinical and nonclinical groups.

	Proportions		Z	*P*-value one tail	Effect size h
	Clinical group	Nonclinical group			
*a:p*	*0.70*	*0.40*	*3.02*	*0.001*	*0.61*
Adj.D	0.24	0.22	0.24	0.406	0.05
Adj.esA	0.14	0.18	−0.55	0.293	−0.11
Adj.esB	0.44	0.48	−0.40	0.344	−0.08
Afr	0.46	0.52	−0.60	0.274	−0.12
An + XY	0.22	0.20	0.25	0.403	0.05
BlendsA	0.02	0.02	0.00	0.500	0.00
BlendsB	0.16	0.22	−0.76	0.222	−0.15
CDI	0.50	0.62	−1.21	0.113	−0.24
CritCont	0.82	0.68	1.62	0.053	0.33
CShBlend	0.14	0.12	0.30	0.383	0.06
DEPI	0.34	0.32	0.21	0.416	0.04
EA	0.34	0.44	−1.03	0.153	−0.21
EgoIndexA	0.32	0.22	1.13	0.130	0.23
*EgoIndexB*	*0.38*	0.56	*−1.80*	*0.036*	*−0.36*
FC:CF + C	0.80	0.68	1.37	0.086	0.28
FMA	0.08	0.12	−0.67	0.252	−0.13
FMB	0.42	0.40	0.20	0.419	0.04
Food	0.16	0.10	0.89	0.186	0.18
Lambda	0.52	0.54	−0.20	0.421	−0.04
*MOR*	*0.22*	0.04	*2.68*	*0.004*	*0.57*
S-%	0.58	0.46	1.20	0.115	0.24
ShShBlnd	0.10	0.10	0.00	0.500	0.00
SumV	0.22	0.24	−0.24	0.406	−0.05
Sumy	0.22	0.34	−1.34	0.091	−0.27
*Wsum6*	*0.34*	0.14	*2.34*	*0.010*	*0.48*
X-%	0.54	0.52	0.20	0.421	0.04

Statistically significant comparisons are highlighted in italics.

Statistically significant differences in the expected direction (higher proportions in the clinical group) were observed in relation to the variables Wsum6, a:p, and MOR. Regarding the EgoIndex variable, there was a statistically significant difference in the opposite direction to the expected one, with higher values in the nonclinical group compared to the clinical group.

### 3.3 Partial correlations while controlling for group

Table 3 presents the partial correlations between the DERS scale scores (in columns) and the selected Rorschach CS dummy score (1 = pathological), while controlling for group membership (clinical vs. nonclinical group).

**Table 3:** Partial correlations between the DERS scale scores (in columns) and Rorschach CS dummy scores (1 = Pathological), while controlling for group membership (clinical vs. nonclinical group).

	Nonacceptance	Goals	Impulse	Awareness	Strategies	Clarity	Total
a:p	−0.10	−0.02	−0.07	−0.15	0.06	−0.14	−0.08
Adj.D	−0.09	−0.03	−0.07	−0.03	−0.01	−0.04	−0.06
Adj.esA	−0.02	−0.02	0.04	−0.06	0.01	0.10	0.01
Adj.esB	−0.12	−0.09	−0.11	0.09	−0.17	−0.13	−0.13
Afr	0.05	−0.08	−0.11	−0.10	0.03	0.02	−0.04
An + XY	0.14	−0.05	0.11	0.07	0.12	0.18	0.14
BlendsA	−0.03	−0.04	−0.01	−0.07	−0.07	−0.06	−0.07
BlendsB	0.12	−0.10	−0.10	0.10	−0.05	−0.06	−0.02
CDI	−0.21^*^	*−0.09*	−0.30^**^	*−0.01*	−0.22^*^	−0.21^*^	−0.25^*^
CritCont	0.07	−0.10	−0.01	0.09	−0.08	0.11	0.01
CShBlend	−0.08	−0.04	0.05	−0.09	0.08	0.06	0.01
DEPI	−0.07	−0.03	−0.05	−0.10	0.06	0.02	−0.03
EA	−0.10	−0.02	−0.13	0.13	−0.08	0.00	−0.06
EgoIndexA	0.02	0.13	0.13	0.20^*^	0.04	0.17	0.15
EgoIndexB	0.05	−0.07	−0.11	−0.09	−0.17	−0.23^*^	−0.14
FC:CF + C	0.03	0.04	−0.03	−0.02	−0.03	−0.07	−0.02
FMA	−0.03	0.12	0.16	0.01	−0.04	0.08	0.06
FMB	−0.01	−0.02	−0.08	0.00	0.07	−0.09	−0.02
Food	0.22*	0.15	0.15	0.06	0.07	0.07	0.16
Lambda	−0.03	−0.04	−0.15	0.02	−0.11	−0.21^*^	−0.12
MOR	0.01	0.05	0.06	−0.10	0.22^*^	0.14	0.10
S-%	0.07	−0.06	0.04	0.06	0.02	0.09	0.05
ShShBlnd	0.00	−0.14	−0.07	0.10	−0.02	0.05	−0.02
SumV	−0.14	−0.09	−0.10	0.09	−0.12	0.11	−0.08
Sumy	0.02	0.14	0.22^*^	0.07	0.10	0.22*	0.17
Wsum6	0.07	0.00	0.10	0.11	0.06	0.20^*^	0.11
X-%	0.21^*^	0.06	0.18	0.25*	0.16	0.19	0.24^*^

^*^p < 0.05; ^**^p < 0.001; statistically significant comparisons are highlighted in italics.

Notably, independently of group membership, there were both positive and negative correlations between the DERS scales and some of the Rorschach CS variables. In particular, the Strategies scale was positively correlated with the Rorschach variable CS MOR; Impulse with SumY; Awareness with high values on the EgoIndex (EgoIndexA) and with X-%; Clarity with SumY and with WSum6 and Nonacceptance with Food and with X-%. The DERS global scale was positively correlated with X-%, the only Rorschach variable that was positively associated with more than one DERS scale.

After controlling for differences as a function of group, the Clarity scale was negatively correlated with the CDI, with low values of EgoIndex (EgoIndexB) and with Lambda. Furthermore, the CDI was also negatively correlated with the Nonacceptance, Impulse, Strategies scales and with global DERS scale.

## 4 Discussion

### 4.1 Comparison of DERS means between the clinical and nonclinical groups

Consistently with our expectations, the adolescents in the clinical group exhibited greater emotional dysregulation than their nonclinical peers, as indicated by the DERS total score. Specifically, it was evident that individuals in the clinical group struggled more with understanding their emotions (Clarity), accepting negative emotions (Nonacceptance), regulating them (Strategies), and controlling their behavior (Impulse). These results reflect the tendency of patients, as readily observed in clinical practice and documented in the literature (Livesley, 2017), to experience an undifferentiated and chaotic mix of emotions, which is a source of suffering and is challenging to describe. Indeed, one of the goals of therapy is to help them to discern their emotional experiences and subsequently “label” them correctly. However, difficulty in accurately naming feelings was not exclusive to adolescents treated in communities, given that the Awareness scale of the DERS, which assesses the ability to recognize and identify emotions, did not yield significant differences between the clinical and nonclinical groups.^1^ It is possible that this competence requires self-reflective skills that adolescents have not yet fully developed, hence the lack of significant differences between the two groups.

Intense and dysregulated emotions are often associated with distorted beliefs about emotions themselves, exacerbating the distress experienced. In these cases, it is not uncommon for patients to self-invalidate their emotional states, repeatedly telling themselves that they should not be feeling these emotions (Livesley, 2017). This internal dialog may evoke secondary feelings of shame, anger, or guilt, which adolescents in therapeutic communities reported more frequently than their un-treated peers. Another characteristic of these patients was their tendency to “fuse” with their emotions (Hayes et al., 1999), viewing emotions as non-transitory, enduring, and identity-defining. It seems that they did not experience an emotion but became the emotion, which could not be modulated. This might explain why adolescents in the clinical group were more convinced than their peers that once emotional reactions were triggered, they could not be modified.

Furthermore, the adolescents in the clinical group reported greater difficulty controlling their behavior when upset, possibly due to a higher degree of emotional lability. This trait is characterized by the tendency to experience intense emotions, even in response to minor events (emotional intensity), leading individuals to react rapidly and in an uncontrolled manner (emotional reactivity; Livesley, 2017). Lastly, there were no significant differences between groups with respect to the Goals scale of the DERS, which assesses difficulty in completing a task when experiencing negative emotions. Specifically, neither adolescents in the clinical group nor those in the nonclinical group reported difficulties in completing goal-directed actions when experiencing negative emotions.^2^

### 4.2 Comparison of Rorschach CS variables across the clinical and nonclinical groups

Among all the Rorschach CS variables examined in our study, only four discriminated between the two groups: MOR, WSum6, displaying a higher number of passive movements than ones (p > a), and low EgoIndex values. For the first three variables mentioned, the clinical group exhibited a significantly higher percentage of cases with pathological scores compared to the nonclinical group. Regarding low EgoIndex values, the percentage of individuals with pathological scores was higher in the nonclinical group.

Based on the results obtained, it is reasonable to assert that the clinical group had more participants with a negative self-representation (MOR), disturbed thinking processes (WSum6), and relationships characterized by dependence on others (p > a). All three of these dimensions are related to emotional dysregulation (Abbate and Porcelli, 2017). Specifically, the functioning of these adolescents appears to be characterized by:

1. A self-representation marked by pessimistic thoughts about the outcomes of their actions, triggering a series of poorly regulated negative emotions (MOR).
2. Relational immaturity, likely stemming from adverse childhood experiences that hindered the development of functional self-regulatory skills, characterized by dependency on others as emotional regulators (p > a).
3. Disturbed thinking processes interfering, among other things, with emotional regulation (WSum6).

In a clinical setting, professionals can use this information as a “warning bell”: if these indicators (especially WSum6 and MOR, which were significantly correlated with Strategies and Clarity scores respectively) are present during the assessment, the clinician may need to assess the presence of some form of emotional dysregulation. Nevertheless, it might be hypothesized that given the nature of the clinical sample and the information available in the literature (Cook et al., 2005), these difficulties may result from early relational trauma, which is often associated with alterations in self-image (Williams, 2009; Van der Kolk, 2015), dysregulated emotions and behaviors (Ricciutello et al., 2012; Livesley et al., 2017), and cognitive difficulties (Cook et al., 2005).

### 4.3 Partial correlations between the DERS scales and Rorschach CS variables, while controlling for group membership

Some of the results obtained were in line with what is found in the literature and thus warrant fresh consideration, while others are counterintuitive and, in some cases, difficult to explain.

First, in our sample, we observed that participants who displayed ideational and emotional pessimism, which is characterized by a sense of helplessness (MOR), perceived their emotions as overwhelming and unchangeable (Strategies). These individuals tended to always see “the glass half empty,” to evaluate relational context with distrust and discouragement, and to apply ineffective and unsystematic logic to their problems (Abbate and Porcelli, 2017). This thinking process increases individuals’ sense of despair, which, in turn, compromises their motivation and increases their feelings of passivity (Livesley, 2017). It seems like these adolescents dwelt in their emotional pain because their all-pervasive pessimism did not help them to explore the situation and alternative strategies for overcoming it. Pessimism made them feel “stuck” in a condition (or emotion) from which they could not see any way out.

Second, those who experienced anxiety due to feeling incapable of dealing with a problematic situation (SumY) tended to lose control over their behavior more easily (Impulse). In stressful situations, these individuals tend to experience decreased attention and ability to make judgments, becoming subordinate to the emotional tension they are experiencing. Such inhibition of appropriate evaluative processes and higher-order cognitive skills (attention, concentration, evaluation, etc.) can lead these individuals (Abbate and Porcelli, 2017) to poorly regulate their behaviors, which is perceived as reduced control over their impulses.

Third, it is observed that participants who were overly self-focused (high Ego Index) or interpreted reality in a distorted manner (X-%) reported not considering their emotions valid, paying no attention to them, and not deeming them worthy of interest (Awareness). Such individuals tend to focus their attention on the negative aspects of their self-image (Abbate and Porcelli, 2017), which will likely be characterized by low self-worth and a lack of legitimacy in expressing their emotions, which are considered wrong and therefore intolerable (Livesley, 2017). Furthermore, these individuals are generally less reflective and may feel trapped in a state of turmoil that they struggle to understand and make sense of. The distorted reading of reality (X-%), on the other hand, may be due to the presence of distorted beliefs that compel individuals to view emotions as harmful and thus to be avoided at all costs.

Fourth, individuals with disturbed thinking processes (WSum6) or pathological levels of anxiety characterized by a sense of helplessness and inability to cope with situations (SumY) are less capable of clearly decoding their emotions (Clarity). It is possible that when pathological levels of fear of failure are reached, it becomes so debilitating that it generates confusion in individuals who can no longer make sense of what they are experiencing (Miers et al., 2011). Additionally, distorted thinking processes may lead to an interpretation of the world (both internal and external) as incoherent, unpredictable, and threatening (Abbate and Porcelli, 2017). The absence of predictability, in turn, increases suffering, because the individual feels overwhelmed by an anguish that cannot be comprehended or processed (Zvolensky et al., 2000).

Finally, those who demonstrated a strong need for dependence on others (Food) had more difficulty accepting their emotions, especially negative ones, which triggered anger, shame, guilt, embarrassment, or weakness (Nonacceptance). Given that the need for dependence is associated with a need to receive care and support, with a difficulty in assertiveness and with a marked sensitivity to loss and rejection (Meyer et al., 2015), we hypothesized that this need for closeness is in conflict with the fear of being rejected by others (Livesley, 2017), generating intense negative emotions that these individuals find unacceptable and from which secondary emotions of embarrassment, guilt, shame, or anger arise. Those who tend to interpret reality in a distorted manner (X-%) also have more difficulties accepting negative emotions (Nonacceptance). In this case, it might be hypothesized that the distorted reading of reality is due to the presence of a dysfunctional belief system that influences the individual’s emotional and relational life. For example, there may be a belief that expressing negative emotions leads to rejection; this belief exposes individuals to secondary feelings of embarrassment, shame, guilt, or anger whenever they experience negative emotions (Gazzillo, 2021).

The study also yielded results contrary to our expectations, which are more difficult to interpret. Specifically, it emerged that, after controlling for differences due to group membership, those with a pathological score for the CDI reported few difficulties in regulating their emotions in responding to the DERS (total scale, Nonacceptance, Impulse, Strategies, and Clarity). The CDI likely reflects chronically limited coping skills, probably stemming from inadequate relational skills that did not develop appropriately during emotional development (Abbate and Porcelli, 2017). Our results seem to suggest that adolescents who are less mature in terms of coping skills also do not report experiencing shame, embarrassment, or guilt in relation to their emotional experiences (Nonacceptance), maintaining control over their behavior (Impulse), and being able to regulate their emotional state (Strategies). Furthermore, they believe that they are not confused about what they feel and can make sense of their emotions (Clarity). A positive CDI is associated with feelings of helplessness and loneliness, a tendency to experience distress that cannot be effectively managed except, for example, by deploying strategies of emotional impoverishment and avoidance (Abbate and Porcelli, 2017). It is possible that adolescents with these characteristics, in order to avoid augmenting their distress or sense of helplessness, avoid confronting their emotions and defensively deny any difficulty in regulating affects.

Additionally, those with low self-esteem who depend entirely on others’ opinions to value themselves (low Ego Index) tend to report less confusion about their feelings and less difficulty in making sense of what they feel (Clarity). The same is true of for those who have an avoidant thinking style and tend to oversimplify stimuli (High Lambda). These individuals defend themselves from emotions by avoiding them and/or avoiding unclear and uncertain situations that trigger them. For example, in the face of an ambiguous stimulus like the Rorschach inkblots, they may enter a state of cognitive dissonance, against which they defend themselves by simplifying the object and not engaging with its complexity. As a result, when faced with the task of saying “what [the blot] might be,” these individuals respond without saying what they actually see (Abbate and Porcelli, 2017). We hypothesized a similar response style for the DERS: indeed, the Clarity scale investigates internal confusion (Gratz and Roemer, 2004; Sighinolfi et al., 2010), and, to protect themselves from the discomfort arising from ambiguity, our adolescents may have activated a “defensive” response style. It is as if, for them, the discomfort resulting from uncertainty can only be resolved by minimizing (or denying) the degree of this uncertainty. Generalizing this way of behaving to daily life situations, these data seem to suggest a mode of functioning that is characterized by low tolerance for ambiguity and uncertain situations.

## 5 Conclusion and future research

The aim of our study was to explore the construct of emotional dysregulation in adolescence using two different diagnostic instruments: a self-report tool specifically designed to assess emotional dysregulation and a performance-based test that implicitly and broadly investigates the personal functioning. Below, we describe some limitations of our work and outline some avenues for future research.

First, it is important to consider the composition of the sample. The adolescents in the clinical group were recruited from therapeutic communities whose patients do not share a specific psychopathological profile, but rather have all been affected by early relational traumas. Hence, the adolescents in the clinical group exhibited a variety of disorders, as also confirmed by the SWAP-200-A, which justified their inclusion in the study, which was not focused on a specific form of psychopathology but on emotional dysregulation. However, this diagnostic heterogeneity may have introduced intervening variables that could be further examined in future studies. Additionally, future research could aim to screen adolescents in the nonclinical group to exclude individuals with psychopathological problems and/or clinically significant levels of emotional dysregulation in the absence of diagnosis and/or treatment.

Second, no information was available regarding the presence of trauma or clearly psychopathological conditions in the nonclinical group. Indeed, there were no exclusion criteria precluding participation in the study; it is therefore possible that some of these adolescents had had traumatic experiences, suffered from psychological symptoms or were receiving treatment in an outpatient setting. Future research could overcome these limitations by including a screening phase for the nonclinical group, aimed at excluding participants who have suffered traumatic experiences and/or who are undergoing psychological treatment and/or who present psychopathological symptoms.

Third, the use of the Rorschach CS in research raises some considerations. This is a diagnostic tool that facilitates comprehensive and in-depth evaluation of personality structure. This result is achieved via a complex interpretation process in which the meaning of each variable is influenced by the values assumed by all the others. It is clear, then, that the interpretation of the test is a complex procedure, making it particularly useful for clinical practice and, at the same time, challenging for research. The interdependence displayed by most of the Rorschach CS indicators enriches the interpretation of individual protocols but is lost when data are collected from numerous individuals. For example, for a finer interpretation of the CS EgoIndex variable, its scores are compared to those obtained on the Fr + rF variable. However, when considering all the data together in a matrix, this comparison is no longer possible, which has repercussions on the interpretation of the results. Indeed, if the nuances of meaning attributed to each variable are lost, this can compromise the interpretation of the relationships observed between the Rorschach variables and the other research data.

Another difficulty concerns the selection of the Rorschach CS variables. Within the Comprehensive System, there are no “official” variables that measure emotional dysregulation or are associated with it. Therefore, although the present selection was guided by the reasoning of expert clinicians and an analysis of the literature, it may be subject to errors.

Despite the limitations described above, our study still provides some food for thought that can be useful for clinical practice and, in particular, for formulating an appropriate therapeutic plan. For example, considering that adolescents with traumatic histories residing in therapeutic communities tend to perceive reality in a distorted manner and have difficulty accepting their negative emotions, a possible goal of community intervention could be to offer young people new relational models that help them to change the underlying belief system that informs their interpretation of events. For example, in their relationships with staff, patients could experience a Significant Other who recognizes their emotions as valid, encourages them to express them, and helps them modulate them. This way, the patient learns not to feel guilty when experiencing negative emotions and modifies the belief that once triggered, an emotion cannot be changed.

Finally, future studies could delve into the counterintuitive data we obtained and could not fully explain.

## Data availability statement

The data analyzed in this study is subject to the following licenses/restrictions: restrictions apply to the availability of these data. Data was obtained from TIARÉ Association for Mental Health and are available on request from the corresponding author with the permission of TIARÉ Association for Mental Health. Requests to access these datasets should be directed to SC, s.cristofanelli@univda.it.

## Ethics statement

The current study was approved by the Ethics Committee of Valle d’Aosta University. The study was conducted in accordance with the local legislation and institutional requirements. The research utilized archive data provided by the Tiarè Association, Mental Health Services. From the data available to us, it is evident that informed consent was requested in written form, in accordance with the Codice Etico AIP (ethical code for research in psychology, Italian Psychological Association) and the provisions of the Italian laws on privacy and data protection (L. 196/2003).

## Author contributions

SC: Conceptualization, Methodology, Writing – original draft, Project administration, Supervision, Writing – review & editing. ST: Methodology, Supervision, Writing – original draft, Writing – review & editing, Data curation, Formal analysis, Software. EC: Methodology, Writing – original draft, Conceptualization. GB: Conceptualization, Methodology, Writing – original draft. FT: Conceptualization, Methodology, Writing – original draft. VV: Conceptualization, Methodology, Writing – original draft. LF: Conceptualization, Methodology, Project administration, Supervision, Writing – original draft, Writing – review & editing.

## Footnotes

^1^We compared the scores of our sample to those obtained in the study conducted by Giromini et al. (2017), adjusted for age by using the equations reported in their contribution. From this comparison, it seems plausible to hypothesize that both our groups (clinical and nonclinical) exhibited unusually high levels of emotional unawareness, even when accounting for difficulties that could be physiologically expected for their age.

^2^We compared the scores of our sample to those obtained in the study conducted by Giromini et al. (2017), adjusted for age by using the equations reported in Contribution. From this comparison, it appears that the adolescents in both the clinical and nonclinical groups displayed levels of emotional dysregulation in terms of difficulty in achieving goals, that may be viewed as developmentally appropriate for their age.
