# Supplementary material for: Exploring emotion dysregulation in adolescence and its association with social immaturity, self-representation, and thought process problems
Source: Front Psychol. 2024 Jul 23;15:1320520. doi: 10.3389/fpsyg.2024.1320520 (PMC11300265; doi:10.3389/fpsyg.2024.1320520)
Supplement: Supplementary file 1 [file Table_1.pdf]

APPENDIX - Rorschach CS variables included in the present study

**Variable name**	**Variable description**	**Variable clinical Meaning**	**Pathological cut-off**
a:p	Active/Passive Movements ratio	Relational immaturity and dependence on others	p>a
Adj.D	Difference between EA and Adjes variables	Level of coping ability regardless of stressors present	< 0
Adj.es	Stable (non-situational) component of the level of stress and discomfort present in the subject	Unusually low level of perceived stress (overestimation of control under stress)<br>Psychological complexity linked to stressors (underestimation of stress control abilities)	<5<br>>9
Afr	Proportion between the sum of the number of answers given to the last three tables and those given to the first seven	Propensity to interpret emotional situations	< .44
An + XY	Somatic concern	Concerns about the vulnerability of the body or its functioning	>2
Blends	Presence of multiple determinants (Colour, movement, chiaroscuro..)	Psychological superficiality/little complexity<br>Tendency to ruminate	0<br>>10
CDI	Coping Deficit Index	Social immaturity due to poor scial and relational competences	>3
CritCont - Critical Contents	Answers including Critical Contents	Disturbed thinking	>0

Variable name	Variable description	Variable clinical Meaning	Pathological cut-off
CShBlend - Color-Shading Blends	Simultaneous presence of determinants of chromatic color and chiaroscuro	confusion or ambivalence regarding feelings	>1
DEPI	Affect and Self-Perception Variables with some variables from other sections	Depressive tendencies	>4
EA	Weighted sum of Human Movement and color	Cognitive and emotional resources	< 4
EgoIndex	Index based on the number of Reflex responses and the presence of two identical objects in the table	Low self-esteem<br><br>Excessive concern about the self	< 3<br><br>>4
FC:CF+C	Ability to modulate emotion	The emotion cannot be modulated because it is very intense and often inappropriate to the context	FC < CF+C
FM	Images of animals engaged in species-appropriate activities	Intrusive ideation (the subject tries to avoid stress, emotions without succeeding)<br><br>Uncontrolled mental activity due to worries, ruminations, stress	<2<br><br>>5
Food	Food responses	Dependency need	>0
Lambda	Semplified thinking	Tendency to oversimplify and low tolerance for ambiguous or uncertain situations; the subject defends himself from the emotion by avoiding conflict situations	>.99

Variable name	Variable description	Variable clinical Meaning	Pathological cut-off
MOR	Sum of responses with Morbid content	Disqualifying vision of the self on both the emotional and the ideational level	>2
S-%	White space used in responses that have Distorted Formal Quality	Presence of strong anger leading to distorted perceptions	>3
ShShBlnd - Shading Blends	Simultaneous presence of multiple types of chiaroscuro determinants	Presence of painful affects constantly present.	>0
SumV	Subtle gradations of chiaroscuro to indicate depth	Negative emotional self-evaluation	>0
SumY	Sum of responses Chiaroscuro Diffuso	Distress or helplessness, often due to strong or moderate stressors	>1
WSum6	Weighted sum of special cognitive signatures	Disturbed thought processes	>17
X-%	Degree to which the images seen are common and appropriate to the spot area	Distorted perception of reality	>.20
